# The association between white blood cell counts and metabolic health obesity among US adults

**DOI:** 10.3389/fnut.2025.1458764

**Published:** 2025-01-17

**Authors:** Zhanbin Li, Zhenyu Yao, Qiaoran Liu

**Affiliations:** ^1^Department of General Surgery, The First Affiliated Hospital of Shandong First Medical University & Shandong Provincial Qianfoshan Hospital, Jinan, Shandong, China; ^2^Key Laboratory of Metabolism and Gastrointestinal Tumor, the First Affiliated Hospital of Shandong First Medical University, Jinan, Shandong, China; ^3^Key Laboratory of Endocrine Glucose & Lipids Metabolism and Brain Aging, Ministry of Education; Department of Endocrinology, Shandong Provincial Hospital Affiliated to Shandong First Medical University, Jinan, Shandong, China; ^4^Shandong Clinical Research Center of Diabetes and Metabolic Diseases, Jinan, Shandong, China; ^5^Shandong Institute of Endocrine and Metabolic Diseases, Jinan, Shandong, China

**Keywords:** blood cell count, WBC, RBC, MHO, NHANES

## Abstract

**Background:**

The correlation between white cell count and metabolically healthy obesity (MHO) remains elusive among obese American adults. This study endeavors to elucidate this association.

**Methods:**

This study enrolled 6,580 obese patients from the National Health and Nutrition Examination Survey (NHANES). Obesity phenotypes were defined by presence/absence of metabolic syndrome components. Weighted multivariate logistic regression analyses were used to assess the association between white cell count and MHO occurrence. Restricted cubic spline analysis characterized dose–response relationships, and stratified analyses explored these relationships across sociodemographic and lifestyle factors.

**Results:**

In this study, MHO prevalence is 11.9% among obese adults. The risk of MHO was inversely correlated with WBC [OR (95%): 0.81 (0.76, 0.86), *p* < 0.001], lymphocytes [OR (95%): 0.56 (0.47, 0.68), *p* < 0.001], monocytes [OR (95%): 0.41 (0.22, 0.75), *p* = 0.004], and neutrophils count [OR (95%): 0.82 (0.76, 0.88), *p* < 0.001]. WBC and neutrophils showed L-shaped associations, while lymphocytes, monocytes, and RBCs had linear patterns. Furthermore, stratified analyses demonstrated blood cell counts consistently predicted MHO risk across subgroups.

**Conclusion:**

In this study, we provide novel insights into the association between blood cell count and the presence of MHO among obese individuals. Blood cell count is an accessible biomarker for dynamically tracking the presence of MHO.

## 1 Introduction

Over the past five decades, the obesity epidemic has soared (1), intimately correlated with the emergence and advancement of metabolic abnormalities, including type 2 diabetes, atherogenic dyslipidemia, metabolic syndrome (MetS), cardiovascular diseases (CVDs), and metabolic dysfunction-related liver disease (MASLD) (2–4). This trend has notably escalated the risks of premature mortality (3) and imposed a considerable burden on public health (5). Despite the clear correlation between obesity and metabolic disorders, a distinct group of obese individuals stands out for their resilience against inflammatory and metabolic complications, labeled as metabolically healthy obesity (MHO) (3, 6). MHO represents a subset of individuals within the broader category of obesity who exhibit a surprisingly favorable metabolic profile, despite their elevated body weight (7). The mechanisms underlying the MHO phenotype remain unclear (6). However, it is crucial to underscore that MHO may also transition to an unhealthy metabolic state over time (8–11). Unfortunately, this important aspect is often overlooked, potentially underestimating the true risk posed by MHO. Meanwhile, the MHO phenotype not only differs from metabolically unhealthy obesity (MUO) but also stands apart from metabolically healthy non-obese (MHNO) individuals. In fact, recent studies have revealed that individuals with MHO may face distinct disease outcomes compared to both MUO and MHNO individuals (7). Individuals with MHO may be at an increased risk of developing cardiovascular disease and certain types of cancer compared to those with MHNO (7, 12). These findings suggest that the MHO phenotype may not be as benign as previously thought, especially when considering these chronic diseases. In the context of the global obesity epidemic, a one-time classification of MHO may not be sufficient. Conversely, overly complex criteria can hinder the ability to make accurate and timely evaluations. Therefore, a simple yet practical indicators that can dynamically track changes of MHO status may be more suitable for clinical applications. By dynamically monitoring and promptly recognizing the potential risks posed by MHO, we can make significant contributions to public health efforts and clinical practice.

MHO exhibits unique biological mechanisms that confer significant advantages over unhealthy obesity. Specifically, individuals with MHO show reduced ectopic fat accumulation, especially in the visceral and liver areas, and increased leg fat deposition. Furthermore, their subcutaneous adipose tissue demonstrates enhanced expandability, preserving insulin sensitivity and *β*-cell function compared to those with MUO (13). Notably, these physiological benefits extend to a significantly reduced profile of inflammatory markers in MHO individuals compared to those with MUO (14–19). This intriguing observation suggests that inflammatory markers, which are fundamental in evaluating and tracking inflammatory states, may serve as promising indicators for assessing MHO status. Among the various inflammatory markers, peripheral white blood cell (WBC) counts, routinely assessed in blood tests, have garnered significant attention due to their extensive utilization in diagnosing systemic inflammatory-related conditions (20). Prior research has consistently demonstrated a robust correlation between peripheral WBC counts and the risk of metabolic syndrome (20–27). This underscores the potential of WBC counts as a reliable indicator for evaluating MHO status. However, despite a previous study that observed significantly lower absolute counts of proinflammatory monocytes in MHO individuals compared to MUO individuals (28), the question remains whether the white cell counts, beyond monocytes, demonstrate any significant correlation with MHO.

To bridge this knowledge gap, we utilize the comprehensive NHANES dataset to conduct a rigorous and thorough analysis of the predictive significance of various blood cell counts in relation to the MHO status. Our primary goal is to identify novel, routinely measurable biomarkers that could significantly contribute to the early detection and ongoing monitoring of MHO, ultimately enhancing our comprehension of this intricate condition.

## 2 Methods

### 2.1 Study population

The NHANES is a nationally representative, ongoing health survey of non-institutionalized U.S. civilians, conducted biennially by the National Center for Health Statistics (NCHS) since 1999. It combines interviews and medical examinations to gather comprehensive data on demographics, socioeconomic status, diet, physiology, and laboratory tests. NHANES has received ethical approval from the CDC’s research ethics review board [NHANES 1999–2004: Protocol #98–12; NHANES 2005–2010; Protocol #2005–06; NHANES 2011–2018: Protocol #2011–17, #2018–01 (Effective beginning October 26, 2017)7], and informed written consent was obtained from all participants. The datasets utilized in the present study are publicly accessible on the official NHANES website^1^.

**Figure 1:**
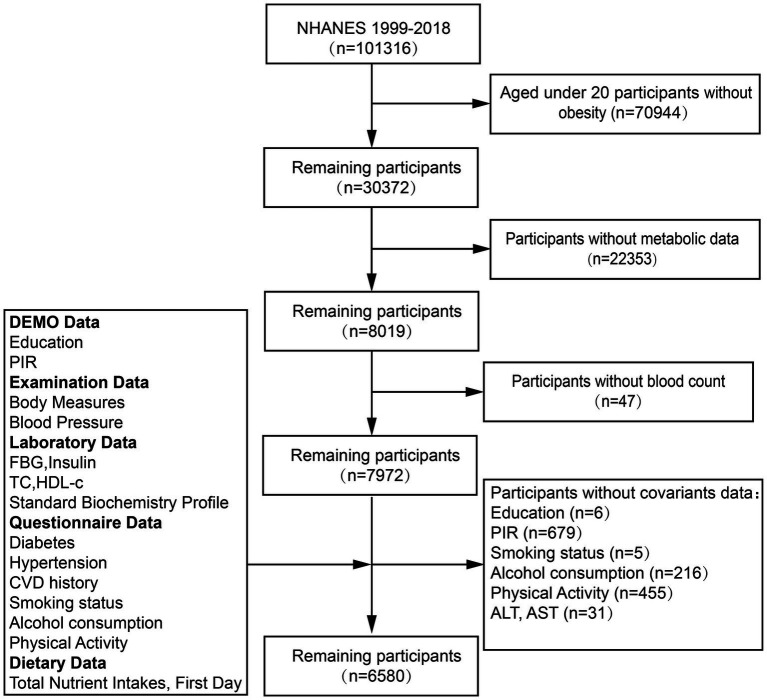
The selection flowchart of the participants.

We conducted this study by utilizing data from 10 consecutive cycles of the NHANES from 1999 to 2018. Participants were excluded for being under 20, BMI < 30 kg/m2, missing metabolic data, or lacking blood cell counts and confounding factor data, ensuring study rigor for MHO biomarker analysis. After rigorous exclusion criteria were applied, 6,580 patients from the NHANES were included in the final analysis. Figure 1 outlines the detailed inclusion and exclusion process.

### 2.2 Obesity phenotype criteria

The diagnostic criteria for obesity phenotypes were mainly based on previous studies (29). Obesity were defined by BMI ≥ 30.0, while metabolic abnormality was assessed based on the presence of specific conditions: (1) SBP ≥ 130 mmHg, DBP ≥ 85 mmHg, or antihypertensive medication use; (2) FPG ≥100 mg/dL or antidiabetic medication use; (3) high-density lipoprotein cholesterol (HDL-C) <40 mg/dL for men and < 50 mg/dL for women; or (4) triglycerides ≥150 mg/dL. Obese participants without metabolic abnormalities were classified as having MHO, whereas those with any abnormalities were designated as having MUO.

### 2.3 Blood cell count assessment

Serum specimens were collected from participants by a phlebotomist at the mobile examination center to assess blood cell count. The Beckman Coulter method, which detects changes in electrical resistance caused by particles suspended in an electrolyte solution, was employed for measurement. This method counts and sizes particles at a rapid rate, enabling the analysis of thousands of particles per second. For this study, we utilized the counter with a unit of 1,000 cells/ul. Further details regarding the laboratory methodology, normal ranges, quality assurance, and data processing are available on the NHANES website: https://wwwn.cdc.gov/Nchs/Nhanes/2013-2014/CBC_H.htm.

### 2.4 Covariates

Confounding factors that were potentially associated with obesity and metabolic status were included in this analysis. Information on age, sex, race, education level, and family income were collected from demographic data. Race was categorized as Mexican American, other Hispanic, non-Hispanic White, non-Hispanic Black, or other race, whereas education level was categorized as less than high school, high school or equivalent, or college or above. Family economic status was determined using the income to poverty ratio (PIR), with three categories: <1.30, 1.31 to 3.50, and ≥ 3.50 (30). Smoking status, alcohol intake, and physical activity were assessed using standardized questionnaires. Participants were categorized into nonsmokers, former smokers, and current smokers, based on their smoking history and habits. Alcohol consumption was classified into three groups: nondrinkers, moderate drinkers (consuming 0.1–27.9 g/day for men and 0.1–13.9 g/day for women), and heavy drinkers (consuming ≥28 g/day for men and ≥ 14 g/day for women). Physical activity was categorized as inactive, active (meeting recommended levels), and insufficiently active (31). The diagnosis of CVD was established through a standardized questionnaire, wherein participants were asked to self-report any physician-diagnosed condition, including Coronary heart disease (CHD), Congestive heart failure (CHF), angina pectoris, Myocardial infarction (MI), or stroke. Affirmative responses to any of these conditions indicate a diagnosis of CVD.

Clinical indicators such as serum Albumin, Alanine aminotransferase (ALT), Aspartate aminotransferase (AST), Blood urea nitrogen (BUN), creatinine (Scr), fasting blood glucose (FBG), HbA1c, triglycerides (TG), total cholesterol (TC), low-density lipoprotein cholesterol (LDL-C), and high-density lipoprotein cholesterol (HDL-C) were measured in the NHANES laboratory, following the relevant standardized protocols. eGFR was calculated using the Chronic Kidney Disease Epidemiology Collaboration Equation (32).

### 2.5 Statistical analysis

Utilizing the powerful R software (version 4.3.1), we conducted a rigorous statistical analysis. Data were categorized into continuous (mean ± SD) and categorical (percentage) variables. For continuous variables, we leveraged the Student’s t-test or the Mann–Whitney *U* test, depending on the data’s distribution. Categorical variables were analyzed using the chi-squared test. Furthermore, stratified obesity phenotypes and baseline characteristics were compared using one-way ANOVA.

To uncover the independent predictive power of blood cell count, we crafted three carefully calibrated multivariate weighted logistic regression models. Model 1 was unadjusted; Model 2 was adjusted for age, race, sex, education level, and family income level; and Model 3 was adjusted for age, sex, race, education level, family income level, ALT, AST, BUN, eGFR, smoking status, alcohol intake, physical activity and CVD history. To visualize the intricate dose–response relationship between blood cell count and the occurrence of MHO, we employed the sophisticated restricted cubic spline (RCS) model. Stratified analyses were performed across various strata, including age (< 30, 30–40, 40–50 or ≥ 50 years old), sex (male or female), race (White, Other Hispanic, Black, Mexican, or Other), education level (less than high school, high school or equivalent, or college or above), family income level (<1.30, 1.31–3.50, and ≥ 3.50), smoking status (current smoker, former smoker or nonsmoker), alcohol intake (heavy drinking, moderate drinking or nondrinking), and physical activity (active, insufficiently or active). The significance level was set at *p* < 0.05. No missing data for covariates and outcomes were recorded in our dataset.

## 3 Results

### 3.1 Baseline characteristics of the participants

Table 1 provides a comprehensive overview of the baseline demographic, lifestyle, and medical profiles of our study participants, categorized into distinct obesity phenotype groups. Our sample had a mean age of 48.07 ± 15.52 years, with a slight majority (53.2%) of women and 46.8% of men. Notably, the prevalence of MHO stood at 11.9%. In comparison to those with MUO, individuals with MHO exhibited a tendency to be younger, of Black ethnicity, female, and had a lower prevalence of comorbidities. Furthermore, our analysis revealed that those with the MHO phenotype had significantly lower levels of WBC, lymphocytes, monocytes, neutrophils, eosinophils, basophils, and RBC counts compared to those with MUO. This finding offers valuable insights into the distinct hematological characteristics associated with different obesity phenotypes.

**Table 1:** Baseline characteristics of participants.

Characteristic^a^	Unweighted			Weighted		
	MHO	MUO	*p*	MHO	MUO	*p*
N (cases)	704	5,876		3298770.36	24513614.86	
age (years)	37.93 ± 13.44	51.19 ± 16.28	<0.001	38.52 ± 13.17	49.36 ± 15.36	<0.001
Gender [Female (%)]	467 (66.3)	3,210 (54.6)	<0.001	2013195.6 (61.0)	12773035.3 (52.1)	<0.001
Race (%)			<0.001			<0.001
Mexican American	128 (18.2)	1,132 (19.3)		336040.5 (10.2)	2242297.2 (9.1)	
Other Hispanic	62 (8.8)	477 (8.1)		251663.1 (7.6)	1174585.4 (4.8)	
Non–Hispanic White	263 (37.4)	2,634 (44.8)		2023382.1 (61.3)	17038217.0 (69.5)	
Non-Hispanic Black	218 (31.0)	1,352 (23.0)		553477.4 (16.8)	2953913.2 (12.1)	
Other Race	33 (4.7)	281 (4.8)		134207.2 (4.1)	1104602.1 (4.5)	
Education (%)			<0.001			0.001
Less than high school	135 (19.2)	1,582 (26.9)		472266.4 (14.3)	4349430.1 (17.7)	
High school	143 (20.3)	1,469 (25.0)		673577.0 (20.4)	6486912.9 (26.5)	
College or above	426 (60.5)	2,825 (48.1)		2152927.0 (65.3)	13677271.9 (55.8)	
PIR (%)			0.216			0.894
<1.30	205 (29.1)	1865 (31.7)		709155.3 (21.5)	5337231.4 (21.8)	
1.31–3.50	280 (39.8)	2,346 (39.9)		1256077.5 (38.1)	9545090.8 (38.9)	
≥3.50	219 (31.1)	1,665 (28.3)		1333537.6 (40.4)	9631292.6 (39.3)	
Smoking status (%)			<0.001			0.002
Current smokers	107 (15.2)	1,077 (18.3)		490063.8 (14.9)	4499663.6 (18.4)	
Former smokers	142 (20.2)	1,673 (28.5)		777355.1 (23.6)	7132354.3 (29.1)	
Non-smokers	455 (64.6)	3,126 (53.2)		2031351.5 (61.6)	12881596.9 (52.5)	
Alcohol consumption (%)			0.005			0.033
Heavy drinking	109 (15.5)	671 (11.4)		590778.4 (17.9)	3285088.0 (13.4)	
Moderate drinking	49 (7.0)	380 (6.5)		258957.9 (7.9)	1700117.0 (6.9)	
Non-drinkers	546 (77.6)	4,825 (82.1)		2449034.1 (74.2)	19528409.9 (79.7)	
Physical activity (%)			<0.001			<0.001
Active	218 (31.0)	1,189 (20.2)		1083949.5 (32.9)	5675854.2 (23.2)	
Insufficiently	186 (26.4)	1,244 (21.2)		930202.3 (28.2)	5811529.1 (23.7)	
Inactive	300 (42.6)	3,443 (58.6)		1284618.6 (38.9)	13026231.5 (53.1)	
CVD [Yes (%)]	17 (2.4)	820 (14.0)	<0.001	75404.9 (2.3)	2886798.0 (11.8)	<0.001
Albumin (g/L)	40.9 ± 3.57	40.99 ± 3.42	0.538	41.37 ± 3.45	41.34 ± 3.28	0.882
ALT (U/L)	24.74 ± 32.16	27.96 ± 19.75	<0.001	25.2 ± 19.85	29.02 ± 19.76	<0.001
AST (U/L)	24.03 ± 22.54	25.74 ± 20.8	0.041	23.73 ± 15.75	25.62 ± 17.4	0.003
BUN (mmol/L)	4.16 ± 1.5	4.97 ± 2.31	<0.001	4.37 ± 1.45	4.97 ± 2.03	<0.001
eGFR (mL/min/1.73 m2)	108.37 ± 21.57	94.51 ± 24.84	<0.001	105.19 ± 19.85	94.55 ± 22.77	<0.001
WBC (1,000 cells/uL)	6.82 ± 2.02	7.23 ± 2.14	<0.001	6.75 ± 1.8	7.27 ± 2.01	<0.001
Lymphocyte (1,000 cells/uL)	2.03 ± 0.62	2.12 ± 0.89	0.01	1.99 ± 0.58	2.1 ± 0.76	<0.001
Monocyte (1,000 cells/uL)	0.52 ± 0.17	0.55 ± 0.23	<0.001	0.52 ± 0.16	0.56 ± 0.22	<0.001
Neutrophils (1,000 cell/uL)	4.03 ± 1.65	4.3 ± 1.66	<0.001	4 ± 1.44	4.35 ± 1.58	<0.001
Eosinophils (1,000 cells/uL)	0.19 ± 0.14	0.21 ± 0.15	<0.001	0.19 ± 0.15	0.21 ± 0.15	0.011
Basophils (1,000 cells/uL)	0.04 ± 0.05	0.05 ± 0.06	<0.001	0.04 ± 0.05	0.05 ± 0.06	0.001
RBC (million cells/uL)	4.67 ± 0.48	4.75 ± 0.51	<0.001	4.74 ± 0.47	4.8 ± 0.48	0.011
Platelet (1,000 cells/uL)	265.34 ± 67.23	254.11 ± 70.37	<0.001	260.08 ± 62.06	255.59 ± 68.76	0.149

a Values are mean (standard deviation) for continuous variables and percentages for categorical variables.

### 3.2 The association of blood cell count with the occurrence of MHO

The results of the logistic regression analysis are presented in Table 2, offer profound insights into the factors associated with the risk of MHO. After rigorous adjustment for relevant covariates, we observed a significant inverse relationship between the risk of MHO and several hematological markers. Specifically, the risk of MHO was negatively correlated with WBC [OR (95%): 0.81 (0.76, 0.86), *p* < 0.001], Lymphocyte [OR (95%): 0.56 (0.47, 0.68), *p* < 0.001], Monocyte [OR (95%): 0.41 (0.22, 0.75), *p* = 0.004], and Neutrophils [OR (95%): 0.82 (0.76, 0.88), *p* < 0.001]. In contrast, no statistically significant association was observed between the risk of MHO and eosinophil or basophil counts. Furthermore, our analysis revealed a negative association between the risk of MHO and RBC count [OR (95% CI): 0.68 (0.52, 0.89), *p* = 0.005]. However, no significant difference was noted in the association between the risk of MHO and platelet count. These findings provide valuable evidence for understanding the complex interplay between hematological parameters and the risk of developing MHO, highlighting potential targets for future research and clinical interventions.

**Table 2:** The association of blood count and MHO in US adults with obesity.

Variables	Model 1		Model 2		Model 3	
	OR (95% CI)	*p*	OR (95% CI)	*p*	OR (95% CI)	*p*
WBC	0.86 (0.82, 0.90)	<0.001	0.79 (0.75, 0.84)	<0.001	0.81 (0.76, 0.86)	<0.001
Lymphocyte	0.77 (0.67, 0.89)	0.001	0.53 (0.44, 0.63)	<0.001	0.56 (0.47, 0.68)	<0.001
Monocyte	0.26 (0.15, 0.45)	<0.001	0.34 (0.19, 0.61)	<0.001	0.41 (0.22, 0.75)	0.004
Neutrophils	0.85 (0.80, 0.91)	<0.001	0.80 (0.75, 0.86)	<0.001	0.82 (0.76, 0.88)	<0.001
Eosinophils	0.36 (0.15, 0.89)	0.027	0.61 (0.26, 1.41)	0.243		
Basophils	0.05 (0.01, 0.32)	0.002	0.09 (0.01, 0.56)	0.01	0.17 (0.03, 1.09)	0.062
RBC	0.77 (0.63, 0.94)	0.012	0.65 (0.50, 0.85)	0.002	0.68 (0.52, 0.89)	0.005
Platelet	1.00 (1.00, 1.00)	0.142				

Multivariate weighted logistics regression models with three models to control for confounding factors. Model 1 was unadjusted; Model 2 was adjusted for age, gender, race, education level and family income level; Model 3 was adjusted for age, gender, race, education level, family income level, smoking status, alcohol consumption, physical activity, CVD history, Albumin, ALT, AST, BUN and eGFR.

### 3.3 The dose-response association of blood cell count with the occurrence of MHO

**Figure 2:**
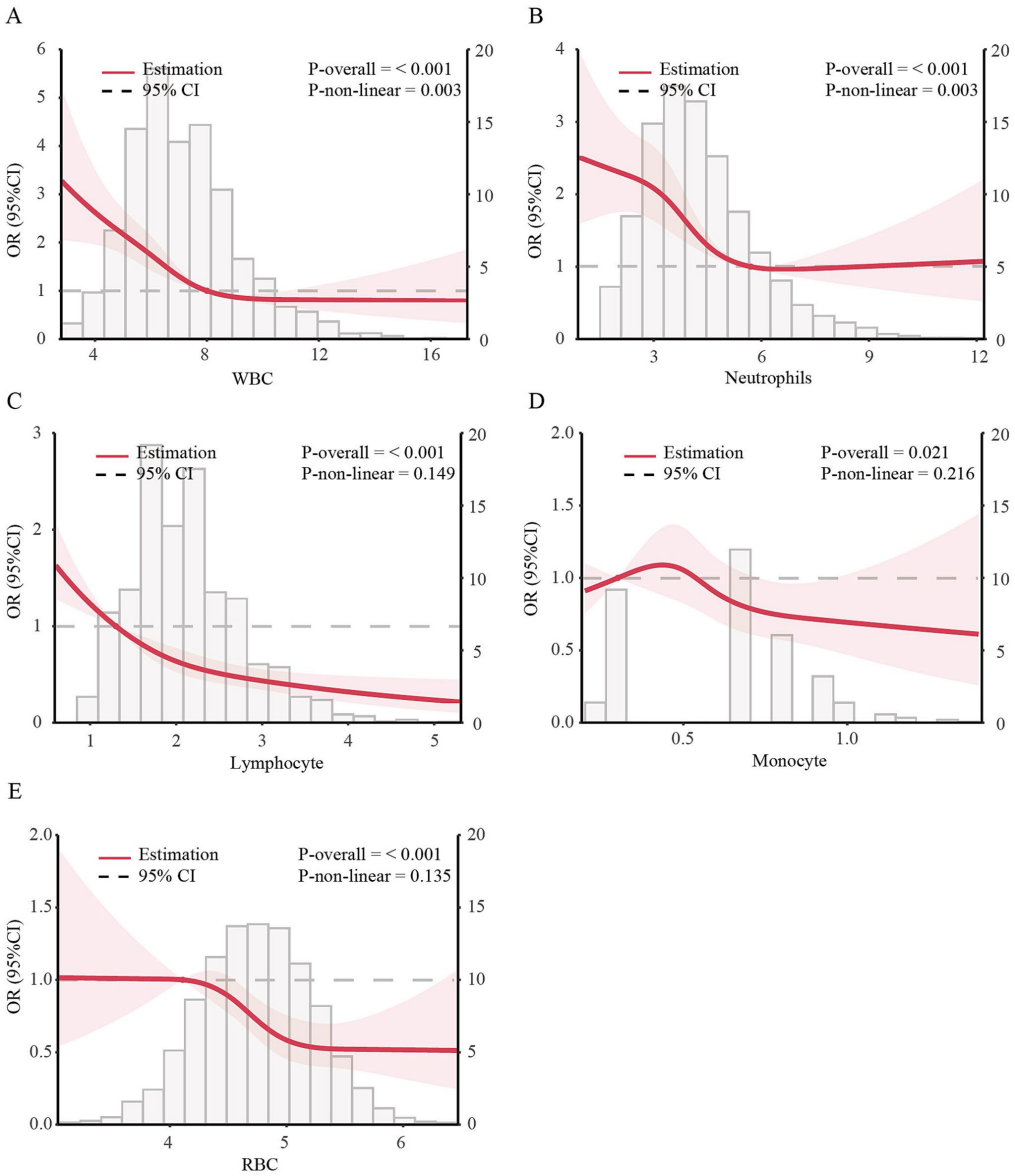
Dose–response associations of blood cell count with incidence of MHO in US obese adults. The dose–response association between incidence of MHO with serum calcium with WBC **(A)**, Lymphocyte **(B)**, Monocyte **(C)**, Neutrophils **(D)**, and RBC **(E)** in US obese adults. ORs adjusted for Model 3 (age, gender, race, education level, family income level, smoking status, alcohol consumption, physical activity, CVD history, Albumin, ALT, AST, BUN and eGFR). Solid lines represent estimates of ORs and dashed lines represent 95% CIs.

Using generalized additive models and restricted cubic spline analysis, we further explored the correlation between WBC, lymphocyte, monocyte, neutrophils and RBC and the risk of MHO. After adjusting for multiple potential confounders, we discovered that the adjusted smoothed plots displayed an L-shaped association of the incidence of MHO with WBC (*P* -nonlinearity = 0.003) and neutrophils count (*P* -nonlinearity = 0.003) (Figures 2A,B). Within the studied dose range, the dose - response relationship between lymphocyte, monocyte, RBC and the risk of MHO were more likely to follow a linear pattern (Figures 2C–E). We discovered that the inflection points of WBC and neutrophils count for the risk of MHO were 8.0 and 5.71, respectively. When WBC count was <8.0, the adjusted OR for MHO [OR (95%): 0.74 (0.67, 0.82), *p* < 0.001] decrease significantly. While neutrophils count was <5.71 mg/mL, the adjusted OR for MHO [OR (95%): 0.75 (0.67, 0.83), *p* < 0.001] decrease significantly. When these counts were exceeded 8.0 and 5.71, there was no association with MHO (Table 3).

**Table 3:** Threshold effect analysis of blood cell count on incidence of MHO in obese patients.

Variables	OR (95% CI)	*p*	*p*-non-linear
WBC	0.81 (0.76, 0.86)	<0.001	0.003
<8.002	0.74 (0.67, 0.82)	<0.001	
≥8.002	0.90 (0.79, 1.03)	0.118	
Neutrophils	0.82 (0.76, 0.88)	<0.001	0.003
<5.706	0.75 (0.67, 0.83)	<0.001	
≥5.706	0.84 (0.69, 1.04)	0.106	
Lymphocyte	0.56 (0.47, 0.68)	<0.001	0.149
Monocyte	0.41 (0.22, 0.75)	0.004	0.216
RBC	0.68 (0.52, 0.89)	0.005	0.135

Logistic regression models were used to estimate OR and 95% CI adjusted for age, sex, race, education level, family income level, smoking status, alcohol intake, physical activity, CVD, serum ALT, AST, BUN and eGFR.

### 3.4 Stratified analyses

The results of the stratified analyses based on age, sex, race, education level, family income level, smoking status, alcohol intake, physical activity and CVD history are shown in Supplementary Figures S1–S5. The ability of blood cell counts to predict the occurrence of MHO among obese patients was consistent across various subgroups. There was a significant interaction between the WBC count, neutrophils count and stratified variables based on race, PIR and alcohol consumption. While a significant interaction between the RBC count and stratified variables based on gender and education. These results underscore the importance of comprehensively evaluating the impact of white cell count on MHO status.

## 4 Discussion

This study analyzed data from 6,580 obese US adults to investigate the relationship between white cell counts and MHO status. Participants with the MHO phenotype had significantly lower WBC, lymphocyte, monocyte, neutrophils, eosinophils, basophils, and RBC counts than those with the MUO phenotype. Notably, a negative association was found between these white cell counts (except eosinophils and basophils) and MHO risk. Subsequently, we uncovering an L-shaped association between the incidence of MHO and WBC and neutrophil counts. Conversely, the dose–response relationship between lymphocyte, monocyte and the risk of MHO followed a more linear pattern. Inflection points were identified at 8.0 for WBC and 5.71 for neutrophils, below which the risk of MHO decreased significantly. Above 8.0 for WBC and 5.71 for neutrophils, no association was observed. Stratified analyses confirmed the predictive role of these blood cell counts in identifying MHO across subgroups. These findings suggest that monitoring these indicators could provide valuable insights for reducing obesity-related metabolic abnormalities.

Our comprehensive study reveals the prevalence of MHO among obese patients in the United States stands at a significant 11.9%. This figure aligns precisely with previous research that showed a steady rise in MHO occurrence, from 10.6% in the 1999–2002 cycles to 15.0% in the 2015–2018 cycles (29). The consistency of our results with these established trends underscores their credibility and reliability. Notably, our analysis uncovers a compelling pattern: individuals with MHO tend to be younger and female, in contrast to those with MUO. This discovery underscores the significant role of demographic characteristics as predictors of MHO. Furthermore, our results echo findings from cross-sectional and longitudinal studies that aging in adults is positively linked to weight gain but negatively associated with metabolic function (33). Moreover, our research identifies significant sex differences in the prevalence of obesity and its metabolic phenotypes. Aligning with previous studies that male children are at a higher risk of developing MUO (34), thus validating the gender disparity in the occurrence of MHO. However, these groundbreaking findings require further exploration to unlock their full potential. Finally, the remarkable diversity in ethnic distribution among our study population offers a unique opportunity to delve into obesity and its related metabolic abnormalities across different racial or ethnic backgrounds. This will help us gain a more comprehensive understanding of the variations and commonalities in obesity issues among diverse populations, ultimately leading to more targeted and effective interventions.

Our study has uncovered a significant negative correlation between diverse blood cell indices and the likelihood of metabolically healthy obesity (MHO), encompassing WBC, lymphocytes, monocytes, neutrophils, and RBC. Prior research has consistently shown that individuals with MHO tend to exhibit a more favorable inflammatory status (15–19, 35). However, to the best of our knowledge, there is a paucity of studies exploring the association between lymphocytes, monocytes, neutrophils, and MHO specifically. Regarding WBC counts, only two studies have investigated their relationship with MHO. For instance, a cross-sectional analysis of 1,889 postmenopausal women from the Women’s Health Initiative Observational Study (WHI-OS) nested case–control stroke study reported a weak association between WBC and MHO (35). While Catherine et al. (14) observed a negative correlation between inflammatory status and MHO status. Notably, our findings echo the first studies. This consistency further underscores the credibility of our results and suggests that WBC counts may emerge as a promising biomarker for assessing inflammatory status and metabolic health in individuals with MHO. Future research is imperative to delve deeper into the underlying mechanisms and clinical implications of this intriguing association.

Intriguingly, our study has uncovered an inverse relationship between RBC count and the risk of MHO. Although previous research has not directly established a link between these two variables, notable studies have provided indirect evidence. One study, which enrolled 110 participants comprising different weight individuals, observed an increase in RBC aggregability among MHO individuals (36). While another study identified a specific RBC fatty acid membrane profile as a potential biomarker for distinguishing MHO in obese patients, especially children (37). These findings highlight distinct RBC membrane lipid characteristics in MHO patients, suggesting their potential role in screening for MHO in pediatric obesity management. These studies indirectly endorse our findings, further validating our results.

Although our research has identified a correlation between blood cell count and MHO, the specific mechanisms underlying this relationship remain elusive and demand rigorous mechanistic inquiries to elucidate the intricate dynamics involved. Individuals with MHO show reduced ectopic fat and enhanced subcutaneous adipose tissue expandability, preserving insulin sensitivity and lowering inflammatory markers compared to MUO (13, 14). This suggests adipose tissue function alterations may be a mechanism. Impaired expandability in MUO leads to altered fat distribution, increasing visceral and liver fat while decreasing leg fat, contributing to insulin resistance and chronic inflammation, and affecting circulating WBC levels.

Obesity and metabolism constitute intricate biological processes that are profoundly influenced by a vast array of diverse factors. In light of this complexity, the accurate identification of MHO demands a comprehensive and multifaceted clinical approach. This approach must incorporate a wide range of assessments, spanning measurements of weight, blood glucose levels, lipid profiles, blood pressure, and a plethora of additional metabolic markers. Meanwhile, MHO phenotype represents a dynamic intermediate stage (11, 38), demanding urgent attention to investigate the long-term dynamic monitoring of alterations in its status. Insights into obesity’s metabolic intricacies pave the way for better prevention and management. Early recognition and monitoring of MHO are crucial to mitigate metabolic abnormalities. Our study introduces blood cell count as a promising accessible indicator for dynamic MHO monitoring, potentially paving the way for further in-depth metabolic assessments and tailored interventions.

To fully appreciate the profound implications of our research findings, it is imperative to acknowledge the limitations inherent in this cross-sectional study. Firstly, we must recognize that causality cannot be definitively established in such a study design. This limitation necessitates the conduct of rigorous cohort studies to confirm our current findings and determine the direction of potential causal relationships. Secondly, cross-sectional studies are inherently vulnerable to the influence of confounding variables, which could potentially introduce bias into our results. Therefore, we must adopt a cautious approach when interpreting our findings and recognize that they require further validation under a variety of conditions and settings. Lastly, while our analysis has focused primarily on the prognostic value of blood cell count in relation to MHO, it is essential to note that this is only a starting point. Further investigation is urgently needed to determine whether changes in blood cell count levels during follow-up periods also serve as predictors of the incidence or progression of MHO. Such longitudinal studies will provide us with a more comprehensive understanding of the dynamic relationship between blood cell count and metabolic health in obese individuals.

In conclusion, we have unveiled a novel complementary approach that capitalizes on blood cell count as a readily accessible indicator for dynamically tracking the presence of MHO among obese individuals. This strategy not only holds promise for an earlier identification of MHO, but also paves a new pathway toward deeper metabolic assessments and tailored therapeutic interventions, thereby advancing the management and prevention of obesity-associated metabolic sequelae.

## Data Availability

Publicly available datasets were analyzed in this study. This data can be found here: https://www.cdc.gov/nchs/nhanes/index.htm.

## Footnotes

^1^ https://www.cdc.gov/nchs/nhanes/index.html
