# Supplementary material for: The association between white blood cell counts and metabolic health obesity among US adults
Source: Front Nutr. 2025 Jan 17;12:1458764. doi: 10.3389/fnut.2025.1458764 (PMC11784339; doi:10.3389/fnut.2025.1458764)
Supplement: Supplementary file 1 [file Data_Sheet_1.docx]

Supplementary Material

# Supplementary Data

Supplementary data include Figures S1-5.

# Supplementary Figures

**
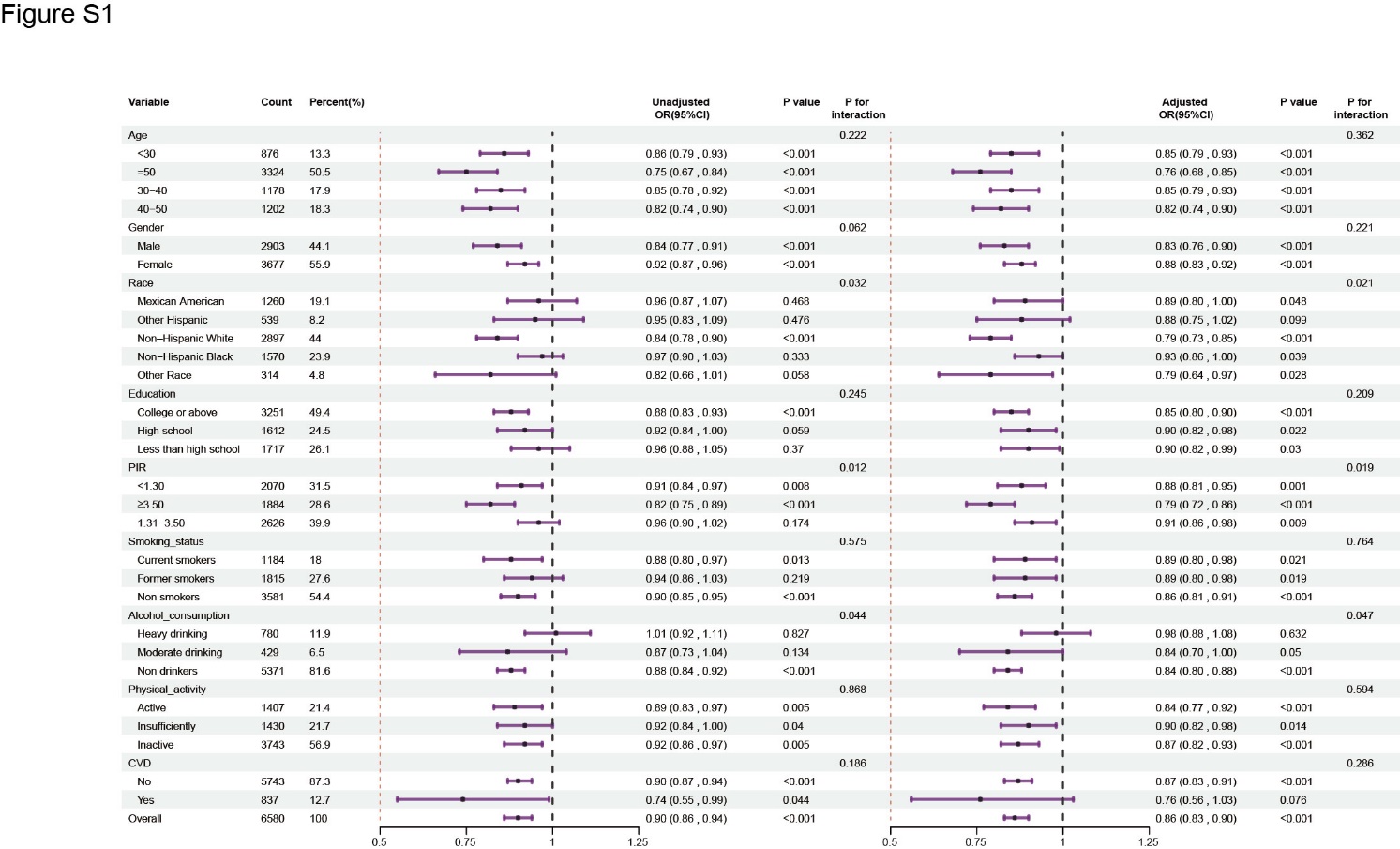
**

**Figure S1:** Forest plot of Stratified analyses of WBC with occurrence of MHO in US obese adults. ORs adjusted for age, gender, race, education level, family income level, smoking status, alcohol consumption, physical activity, CVD history, Albumin, ALT, AST, BUN and eGFR.


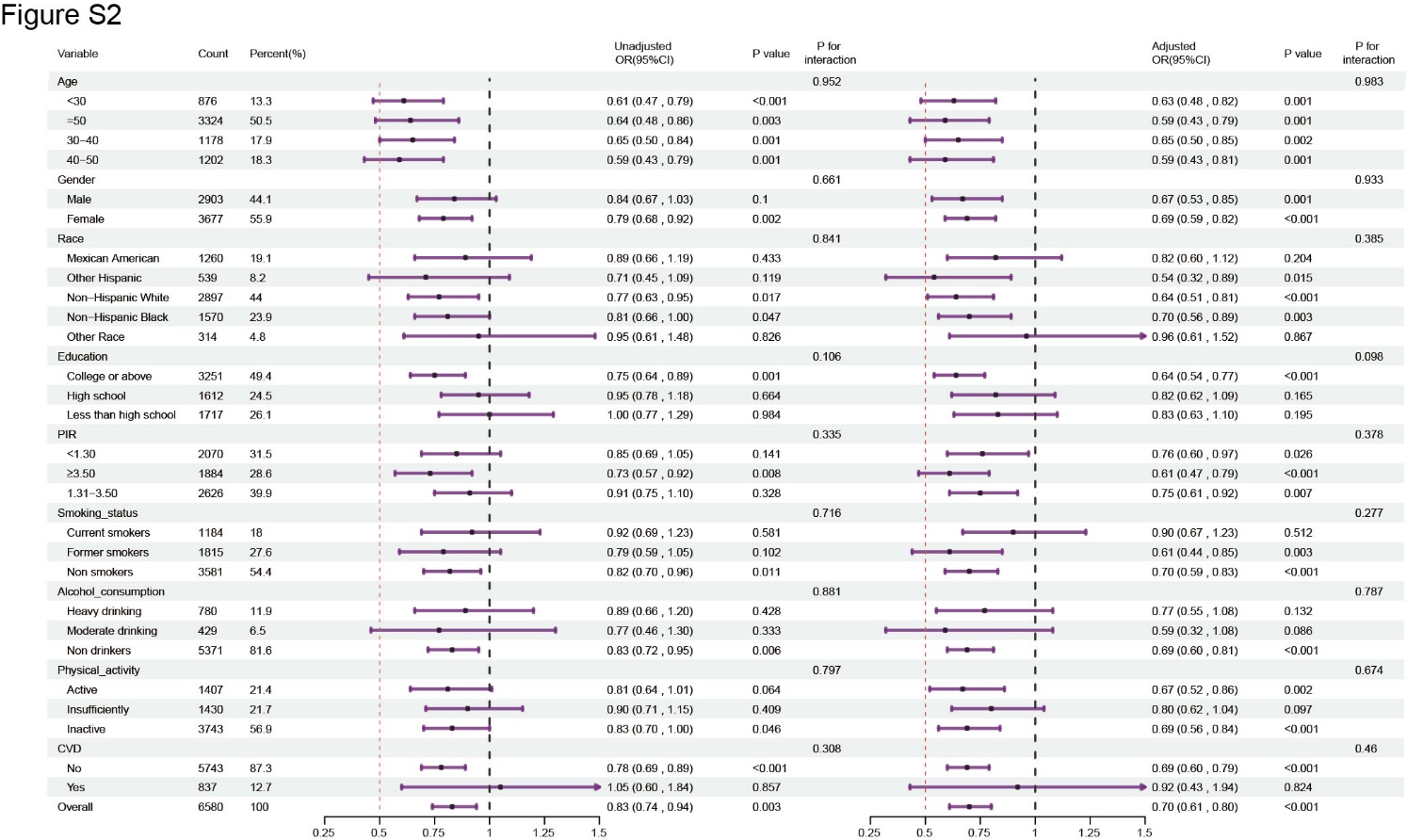


**Figure S2:** Forest plot of Stratified analyses of lymphocyte with occurrence of MHO in US obese adults. ORs adjusted for age, gender, race, education level, family income level, smoking status, alcohol consumption, physical activity, CVD history, Albumin, ALT, AST, BUN and eGFR.


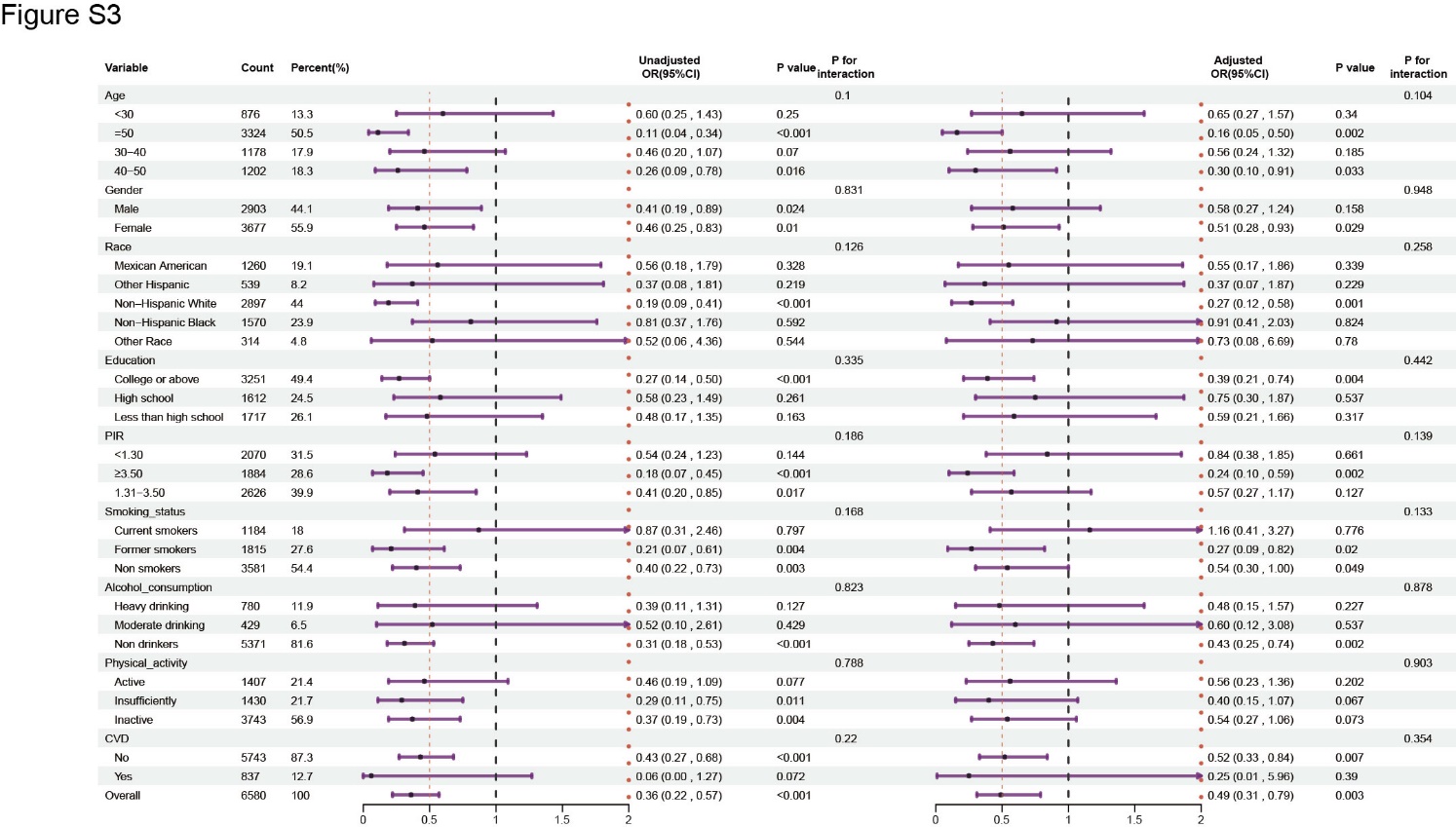


**Figure S3:** Forest plot of Stratified analyses of monocyte with occurrence of MHO in US obese adults. ORs adjusted for age, gender, race, education level, family income level, smoking status, alcohol consumption, physical activity, CVD history, Albumin, ALT, AST, BUN and eGFR.


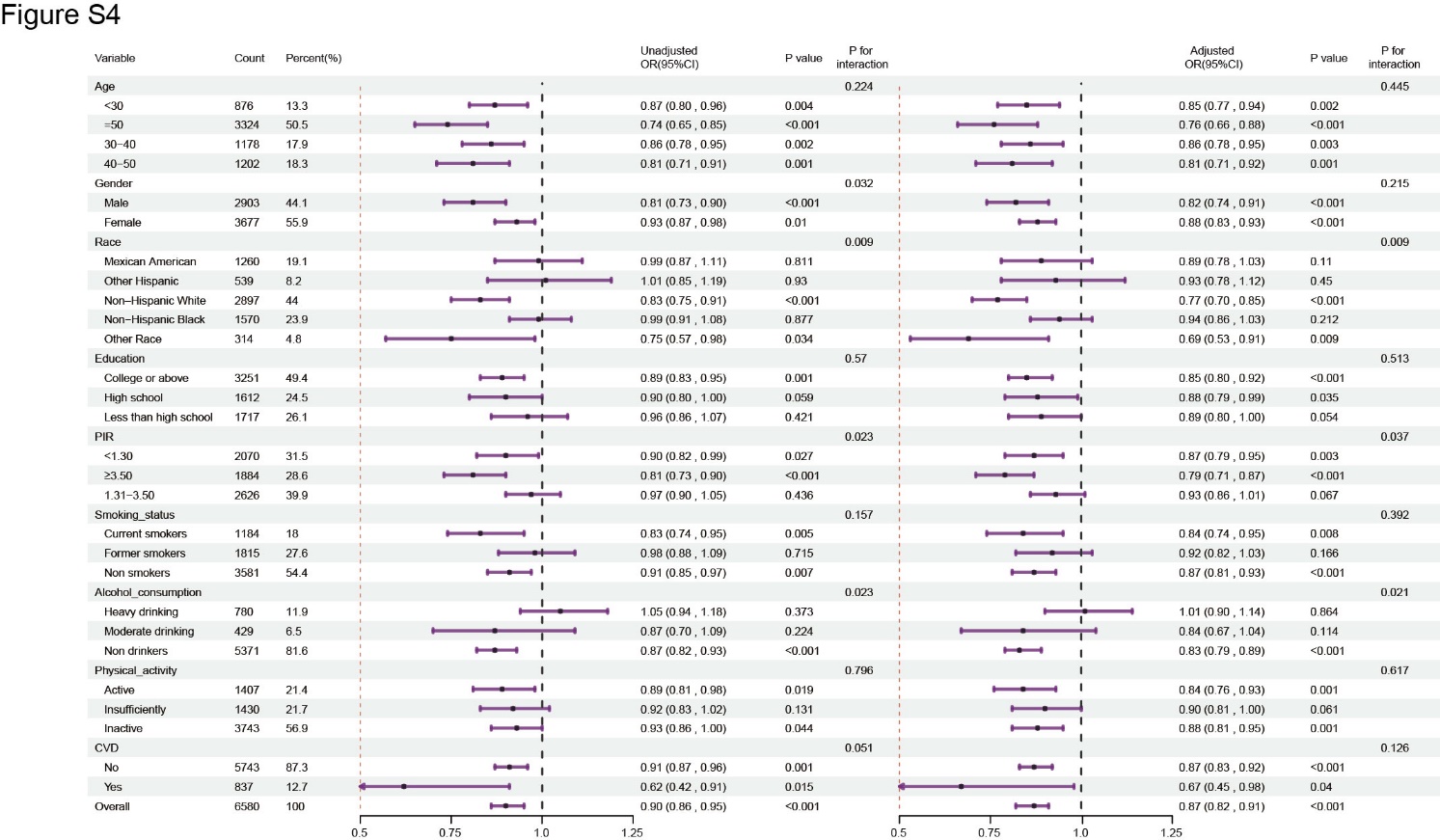


**Figure S4:** Forest plot of Stratified analyses of neutrophils with occurrence of MHO in US obese adults. ORs adjusted for age, gender, race, education level, family income level, smoking status, alcohol consumption, physical activity, CVD history, Albumin, ALT, AST, BUN and eGFR.


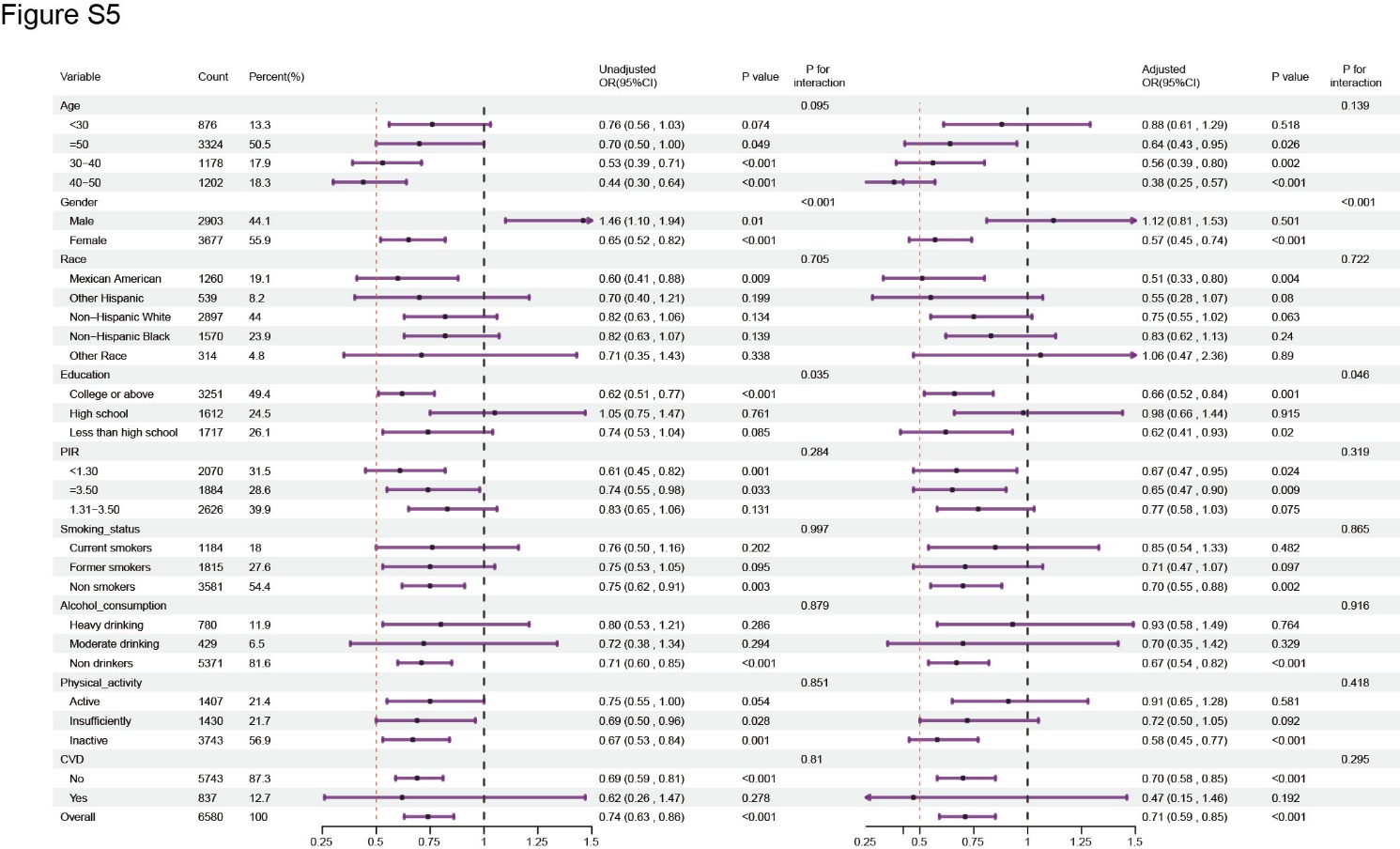


**Figure S5:** Forest plot of Stratified analyses of RBC with occurrence of MHO in US obese adults. ORs adjusted for age, gender, race, education level, family income level, smoking status, alcohol consumption, physical activity, CVD history, Albumin, ALT, AST, BUN and eGFR.
